# A robust sound perception model suitable for neuromorphic implementation

**DOI:** 10.3389/fnins.2013.00278

**Published:** 2014-01-17

**Authors:** Martin Coath, Sadique Sheik, Elisabetta Chicca, Giacomo Indiveri, Susan L. Denham, Thomas Wennekers

**Affiliations:** ^1^Cognition Institute, Plymouth UniversityPlymouth, UK; ^2^Faculty of Health and Human Sciences, School of Psychology, Plymouth UniversityPlymouth, UK; ^3^Institute of Neuroinformatics, University of Zurich and ETH ZurichZurich, Switzerland; ^4^Faculty of Technology, Cognitive Interaction Technology – Center of Excellence, Bielefeld UniversityBielefeld, Germany; ^5^Faculty of Science and Environment, School of Computing and Mathematics, Plymouth UniversityPlymouth, UK

**Keywords:** auditory, modeling, plasticity, information, VLSI, neuromorphic

## Abstract

We have recently demonstrated the emergence of dynamic feature sensitivity through exposure to formative stimuli in a real-time neuromorphic system implementing a hybrid analog/digital network of spiking neurons. This network, inspired by models of auditory processing in mammals, includes several mutually connected layers with distance-dependent transmission delays and learning in the form of spike timing dependent plasticity, which effects stimulus-driven changes in the network connectivity. Here we present results that demonstrate that the network is robust to a range of variations in the stimulus pattern, such as are found in naturalistic stimuli and neural responses. This robustness is a property critical to the development of realistic, electronic neuromorphic systems. We analyze the variability of the response of the network to “noisy” stimuli which allows us to characterize the acuity in information-theoretic terms. This provides an objective basis for the quantitative comparison of networks, their connectivity patterns, and learning strategies, which can inform future design decisions. We also show, using stimuli derived from speech samples, that the principles are robust to other challenges, such as variable presentation rate, that would have to be met by systems deployed in the real world. Finally we demonstrate the potential applicability of the approach to real sounds.

## 1. Introduction

Neurons in sensory cortex are highly adaptive, and are sensitive to an organism's sensory environment. This is particularly true during early life and an epoch known as the “critical period” (Zhang et al., 2001; Insanally et al., 2009). For many organisms sounds of ecological importance, such as communication calls, are characterized by time-varying spectra. Understanding how to build auditory processing systems that can cope with time-varying spectra is important. However, most neuromorphic auditory models to date have focused on distinguishing mainly static patterns, under the assumption that dynamic patterns can be learned as sequences of static ones.

One strategy for devices that implement artificial sensory systems is to emulate biological principles. Developing this approach holds out the hope that we might be able to build devices that approach the efficiency and robustness of biological systems and, in doing so, new insights in to neural processing might be gained. If, as is widely believed, the perception of complex sensory stimuli *in vivo* is based upon the population response of spiking neurons that are tuned to stimulus features then important questions arise, including “what are these features, and how do they come in to existence?” The situation for artificial auditory perception is complicated by the fact that the way in which sounds are represented in mammalian auditory cortex is not well understood, and neither are the neural mechanisms underlying the learning of dynamic sound features.

Neural mechanisms thought to underlie, for example, sensitivity to frequency sweeps include differential latency between excitatory inputs (Razak and Fuzessery, 2008), or excitatory and inhibitory inputs (Razak and Fuzessery, 2010), and asymmetric inhibition (Zhang et al., 2003; Razak and Fuzessery, 2009), all of which have been shown to correlate with sweep direction and/or rate preference. However, these studies have focussed primarily on local neural mechanisms (Ye et al., 2010) whereas anatomical studies of the auditory system reveal widespread lateral connections and nested recurrent loops, and in many cases feedback connections outnumbering feed-forward ones (Friston, 2005).

We have demonstrated previously that it is possible to address the problem of sensitivity to dynamic stimuli, including but not limited to frequency modulated (FM) sweeps, with a biophysically plausible model of auditory processing (Coath et al., 2010). We have validated the model with a real-time physical system implemented using neuromorphic electronic circuits (Sheik et al., 2011). However, neither of these studies has investigated the robustness of the system to stimuli that exhibit variation, either in spike pattern, or presentation rate, or to the order of similar stimuli when sets of stimuli are presented continuously. In addition, the spectro-temporal patterns used as stimuli in these earlier studies are not derived from, or related to, those found in natural sounds such as speech, or other communication calls of animals. All of these considerations are important if the principles involved are to be implemented in artificial sensory systems that can be deployed in realistic environments.

In the present paper we provide evidence that the approach first presented in Sheik et al. (2011) is suitable for “real-world” deployment in that we extend the hardware results to an investigation of responses to “noisy” stimuli. We also present results from a software simulation that replicates the hardware as closely as possible using stimuli derived from speech and presented continuously at different rates. Robustness to both of these types of stimulus variation is a necessary condition for any practical system. Finally we predict the results from networks with comparable architectures trained on real world stimuli. This approach is useful in that it provides guidelines that can be used to inform the design of more complex neuromorphic processing systems that could be implemented in the future.

## 2. Methods

### 2.1. Network

#### 2.1.1. Schematic

**Figure 1:**
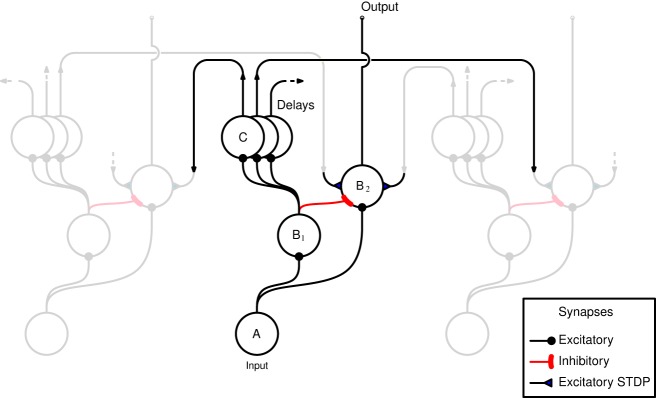
**Schematic representation of the network as implemented in hardware and software**. Neurons are arranged in groups representing positions on the tonotopic axis. Three channels only are shown, and of these only the central channel is labeled for clarity. The two flanking channels are shown dimmed to illustrate how the channels interconnected by delayed excitatory projections that terminate in plastic synapses. The two populations of B neurons receive input from the same A neurons within the same channel. B_2_ neurons are excited by B_1_ neurons from outside their own channel.

A schematic representation of the network, as implemented in both hardware and software, is shown in Figure 1. The horizontal axis in the figure represents the tonotopic arrangement of the auditory system, divided in to a number of frequency channels representing positions on the basilar membrane. The pattern of spiking in the A neurons thus represents the output of an artificial cochlea (Chan et al., 2007). Three channels only are shown in Figure 1, the central channel is labeled and the two flanking channels are shown “dimmed” to illustrate how the neurons within each channel are laterally connected to other channels. The hardware implementation and the software simulation both use 32 tonotopic channels. Where real stimuli are processed (see section 2.1.5) the cochlea uses a linear gammatone filter bank, followed by half wave rectification and low pass filtering to simulate the phase locking characteristics of auditory nerve firing, and center frequencies ranging from 50 to 8000 Hz equally spaced on the Equivalent Rectangular Bandwidth scale (Glasberg and Moore, 1990).

The input neuron A, at each tonotopic position projects to a B_1_ and a B_2_ neuron in the same channel *via* excitatory synapses. The output of the network is taken to be the activity of the B_2_ neurons. This activity is derived from the input, but controlled by excitatory and inhibitory projections from B_1_ neurons. However, the excitatory B_1_ → B_2_ projections originate *only* from other tonotopic channels, these connections exhibit distance dependent propagation delays, and terminate with plastic synapses which are the loci of Spike Timing Dependent Plasticity (stdp) (see section 2.1.2). Each B_1_ neuron is connected to a number of B_2_ neurons *via* these delayed connections that have a fan out of 14 neurons on either side. The learning rule implemented at the synapses associated with these connections (shown as filled triangles in Figure 1) ensures that the B_2_ neurons are active only if there are coincidences between spikes within the channel and delayed spikes from other channels; it is this feature that allows the network to learn dynamic spectro-temporal patterns. The units marked C represent the delays in the B_1_ → B_2_ connections which are implemented differently in hardware and software, see sections 2.1.3 and 2.1.4.

#### 2.1.2. Spike timing dependent plasticity

Plasticity in both the hardware and software networks is implemented in each of the B_1_ → B_2_ synapses in the form of an stdp-like model of synaptic plasticity described fully in Brader et al. (2007). In the absence of activation, the synaptic weight, or efficacy, drifts toward one of two stable values, 0 or 1; and although it can take on other values, it is bounded by these two values and stays constant at one of them unless further learning events occur. This has the advantage of preventing instabilities in the adaptation, such as the unbounded growth of connection strengths.

#### 2.1.3 Hardware implementation

The first set of results presented in section 3.1 were obtained using a hybrid analog /digital hardware implementation of the network model which consists of a real-time, multi-chip set-up as described in Sheik et al. (2011). Three multi-neuron spiking chips and an Address Event Representation (aer) mapper (Fasnacht and Indiveri, 2011) are used connected in a serial loop. The multi-neuron chips were fabricated using a standard AMS 0.35 μm CMOS process.

The hardware does not directly support propagation delays between neurons. To overcome this limitation, long synaptic and neuronal time constants are exploited, which due to the variability in hardware have a range of values (Sheik et al., 2012). Given that the weights associated with the synapses of a neuron are strong enough to produce a single output spike, the time difference between the pre-synaptic spike and the post-synaptic spike is considered equivalent to a propagation/transmission delay. Therefore, every projection in the model that requires a delay is passed through an additional neuron, referred to as a delay neuron. The delay neurons are labeled C in Figure 1.

**Figure 2:**
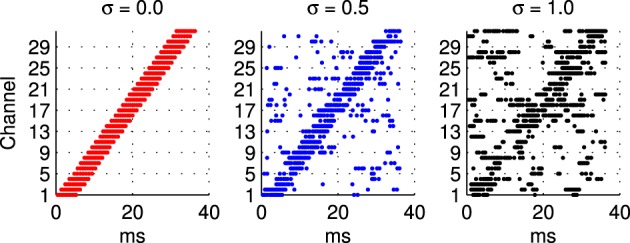
**Illustration of sample spike patterns used as probe stimuli in the hardware experiments described in section 2.1.3 to investigate the robustness of the network response to variability in the probe stimulus**. In all cases these patterns of spikes are prepared off-line using a model integrate and fire neuron, a 5.5 ms current pulse, and a noise current that extends over the whole stimulus period. From left to right the ratio between the noisy current the current pulse used to generate the spike in a channel increases from 0 to 1. The range illustrated is greater than that used in the experiments where the highest level of noise is σ = 0.45.

***2.1.3.1 Frequency modulated stimuli***. Trials with the hardware network were conducted with stimuli representing Frequency Modulated (fm) sweeps. These were prepared off-line by injecting current in to integrate and fire neurons. A current pulse of duration 5.5 ms is used in each channel in turn to generate the burst of input spikes representing the activity of the A neurons (see Figure 1) when presented with a frequency modulated stimulus. In order to evaluate the robustness of the network response to stimulus variation, or noise, an additional noisy current signal is added to this injection current used to generate the input spikes as illustrated in Figure 2. Noise is generated from an Ornstein Uhlenbeck (ou) process with a zero mean using the forward Euler method (Bibbona et al., 2008). We define the noise level, σ as the ratio between the standard deviation of the ou process and the magnitude of the actual noise free current signal used to generate the spikes.

***2.1.3.2. FM sweep trials and analysis***. Trials for the hardware and software versions of the network consisted of two parts; first the *exposure* phase, using the exposure stimulus (es), followed by a *probe* phase using a number of different probe stimuli (ps) presented many times. During the exposure phase the learning rule forces the weight, or efficacy, of each B_1_ → B_2_ plastic synapses to either one or zero; this effects a pattern of stimulus driven connectivity. The selection by the learning rule of only a few high efficacy connections is the origin of the difference in response characteristics between the B_1_ and the B_2_ neurons (Sheik et al., 2011).

The method adopted in the first set of experiments (section 3.1) using the hardware implementation of the network and FM sweep stimuli was the same as that described in Sheik et al. (2011). We reset the neurons to their resting state at the beginning of each es and the plastic synapses to their “low” state, that is with effectively null synaptic efficacy. Input patterns were presented 30 times over a period of 3 seconds during which time the network “learns.” We then measured the response of the exposed network by probing with a set of ps that consisted of linear frequency sweeps with different velocities; during each of these presentations the number of spikes in the *B*_2_ neurons was recorded. Stimuli representing each of the 10 sweep rates were presented 100 times for each noise level during the probe phase. These results were used to determine the Stimulus Specific Information (SSI) as described below.

***2.1.3.3. Stimulus Specific Information***. As artificial sensory systems become increasingly complex it will become increasingly important to make principled decisions about their design. In the majority of cases choices will have to be made where the detailed neurobiological data is incomplete or difficult to interpret. This inevitably leads to a requirement to quantify the performance of the network (for comparison with *in vivo* data, and to guide choices of architecture, learning rule, etc.) where no clear guidance is available from physiology.

A measure that has been used to characterize neuronal acuity is the Stimulus Specific Information (ssi) which is a formalization of the intuitive view that a stimulus is well encoded if it produces an unambiguous response; that is a response that is associated with a unique, or very small number of, stimuli. Where this is true the stimulus is readily identified when one of these responses, or a response in the correct range, appears (Butts and Goldman, 2006). This characterization has the advantage of not being dependant on the design or performance of a classifier. The specific information of a response *i*_*sp*_(*r*) given a set of stimuli Θ can be written:

$$i_{sp}(r)=-\sum_{\Theta }p(\Theta )\log_{2}p(\Theta )+\sum_{\Theta }p\left(\Theta |r\right){\text{log}}_{2}\;p\left(\Theta |r\right)$$

where it is defined in terms of the entropy of the stimulus ensemble, and that of the stimulus distribution conditional on a particular response. This makes the *i*_*sp*_(*r*) a measure of the reduction in uncertainty about the stimulus Θ gained by measuring particular response *r*. Thus the value of the *i*_*sp*_(*r*) is high for unambiguous responses and low for ambiguous responses. The ssi is simply the average specific information of the responses that occur when a particular stimulus, Θ, is present:

$$i_{\text{SSI}}(\Theta )=\sum_{r}p\left(r|\Theta \right)i_{sp}(r)$$

We show that the performance of the network can be characterized by the ssi which combines features of the tuning curve, where information is encoded in the rate of response, and of the Fisher Information where the high-slope regions of the tuning curve are the most informative (Butts and Goldman, 2006).

***2.1.3.4. Receiver Operating Characteristics***. A Receiver Operating Characteristic (roc) can be used as a measure of performance of classifiers (Fawcett, 2006). ROC graphs have their origin in signal detection theory but are also popular in other fields, including the evaluation and comparison of machine learning algorithms. The output from the network can be interpreted as a binary classifier if we designate the Exposure Stimulus as the target for identification by setting a detection threshold. The roc is then a graph of the False Positive Rate (fpr) against the True Positive Rate (tpr) for all values of the detection threshold. The fpr is simply the ratio between the number of stimuli of the target class correctly identified (True Positives, tp) and the total number of stimuli identified as belonging to this class (Positives, p):

$$\text{TPR}=\frac{\text{TP}}{\text{P}}$$

Likewise, the fpr is the ratio between the the number of stimuli incorrectly identified as belonging to the target class (False Positives, fp) and the total number of stimuli identified as not belonging to this class (Negatives, n):

$$\text{FPR}=\frac{\text{FP}}{\text{N}}$$

The roc curve is a two-dimensional visualization of the system's potential as a classifier. We also make use of a common method to reduce this to a single scalar value, that is to calculate the area under the roc curve, abbreviated auc; this is achieved by adding the area of successive trapezoids (Fawcett, 2006). The Area Under Curve (auc) is used to quantify the relative overall ability of the network to discriminate between the two classes of stimuli; that is those that match the class of the Exposure Stimulus and those that do not. This method has been widely used in the characterization of classifiers and is believed to perform very well (Fawcett, 2006). In all cases the auc will be between 0.5, representing a network that will not function as a classifier, and 1.0 which represents a perfect classifier at all thresholds. Although useful, unlike the ssi this ignores the information present in the response concerning any of the other six classes.

#### 2.1.4. Software implementation

A second set of results, presented in section 3.2, were obtained using a network implemented in custom “C” code closely based on the hardware implementation. The learning rule implemented is also the same as in the hardware implementation (see section 2.1.2). In these software simulations of the hardware implementation the lateral, or B_1_ to B_2_, projections exhibit distance dependent delays that cover the same range of values as the hardware network, however these delays were implemented in a queued data structure whereas in the hardware these delays are implemented by exploiting variability of time constants that result from the fabrication of the chip (Sheik et al., 2011, 2012). Beside this difference the software model was designed to be close to the hardware implementation in order to allow for reliable predictions of the hardware's learning and recognition capabilities. Because the hardware operates in real biological time, use of an emulated software version allowed us to run a large number of tests, which would have been impossible in hardware.

***2.1.4.1. Stimuli derived from speech***. The stimuli used in these experiments using the software network were derived from speech and represent the formant tracks of a set of English words. Formants are peaks in the frequency response of sounds caused by resonances in the vocal tract. These peaks are the characteristics that identify vowels and in most cases the two first formants are enough to disambiguate a vowel. This approach was chosen as it results in stimuli that increase the complexity and realism from the single, and double, FM sweeps used in the first set of experiments.

A vocabulary of seven words was chosen: *And, Of, Yes, One, Two, Three, Four* and three examples of each were recorded using a male speaker (the first author). Seven words were chosen because they exhibit a variety of vowel sounds, and hence their formant tracks exhibit a range of spectrotemporal correlations, also they are monosyllabic and (almost) free of diphthongs. The formant tracks of these words exhibit spectrotemporal correlations, for example changes in frequency over time and maxima at two different spectral positions at the same time, that we have shown can be learned by the network—there is more on the mechanism of this learning in section 3.1.

**Figure 3:**
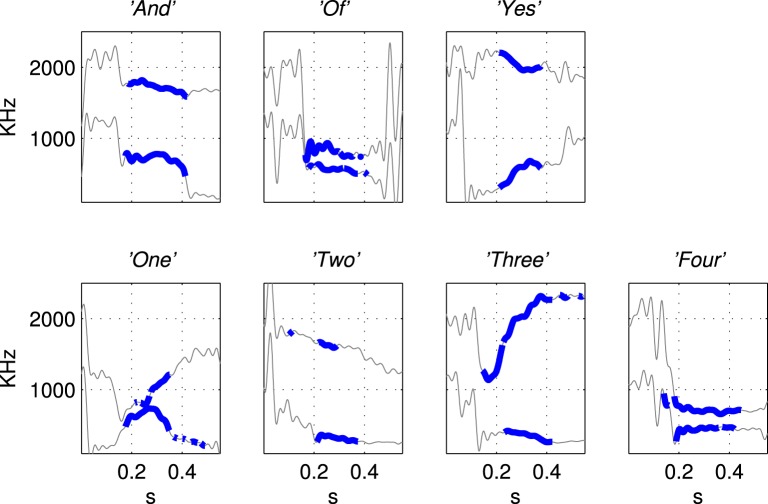
**Illustration of the derivation of simplified stimuli consisting of the first and second formant tracks for the seven words extracted using Linear Predictive Coding**. The words were *“And”, “Of”, “Yes”, “One”, “Two”, “Three”, “Four”* as labeled in titles of subfigures. The thin line segments (in gray) are the parts of the sound files that were silent or contained consonants. Formant tracks of vowels, shown in thicker blue line segments, were smoothed and down-sampled to produce the patterns of current injection that were a highly simplified representation of the speech stimuli, see Figure 5.

**Figure 4:**
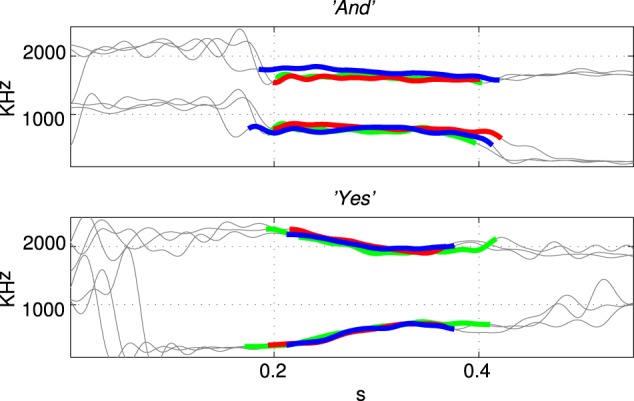
**Formant tracks extracted using lpc for three examples of two different stimuli “And” and “Yes.”** Sections of the individual stimuli corresponding to vowels are indicated by thicker red, green, and blue segmemts this Figure clearly shows that there is some variation in the formant tracks among sets of stimuli of the same class even when recorded from a single speaker.

The first and second formant tracks of these seven classes were extracted using lpc which yields position (frequency) and magnitude parameters for formants (Ellis, 2005). The results are shown in Figure 3 in which parts of the stimulus indicated with a thicker line (in blue) are those with an lpc magnitude of greater than 15% of the maximum value indicating the position of the vowel. The thin line sections (in gray) correspond to the parts of the sound files that were silent or contained consonants. Figure 4 shows how the three examples of each word have formant tracks that are comparable. For clarity only two of the seven words are shown in Figure 4 and the extracted formant tracks highlighted using thicker colored lines as in Figure 3.

**Figure 5:**
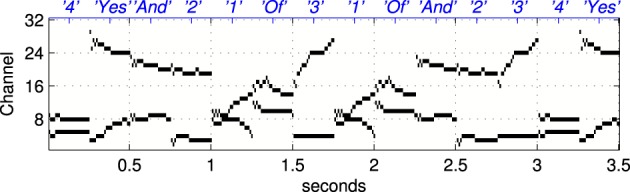
**An example of a stimulus sequence, or “sentence,” used as a Probe Stimulus (ps) for the second set of experiments in software simulations**. The stimulus is a concatenation of simplified formant tracks drawn from the set of words illustrated in Figure 3. The labels on the upper abcissa (in blue) show the stimulus class. Each “word” is arranged to be 250 ms long, hence the presentation rate in the trials referred to as “normal” is 4 stimuli per second, see section 2.1.4.

The formant tracks were then smoothed and down-sampled to produce the patterns of current injection that were a simplified representation of the stimulus, see Figure 5. These patterns of current injection derived from the formant tracks are stored as 32 × 25 binary patterns used as inputs to the network simulation, with each of the 32 rows representing a frequency channel and each of the 25 columns representing temporal bins of 10 ms. Thus with each monosyllabic word occupying 250 ms presentation at the ‘normal’ or 100% presentation rate is 4 stimuli per second, a realistic rate for speech. We use the same stimuli presented at other rates (60, 150, 200% of the normal rate of 4 stimuli per second) to investigate robustness to time warping, see section 2.1.4.

Random concatenations of the 21 stimuli produced simplified, formant based representations of nonsense sentences of the type *“three and one of four two four yes and one of two three yes one”* etc, an example of which is shown in Figure 5. The sentences were arranged to contain equal numbers of each stimulus and were presented during the exposure phase without gaps.

***2.1.4.2. Formant track trials and analysis***. In the exposure phase of the second set of experiments reported in section 3.2 the network was exposed to 20 repetitions (5 s) of all three examples of a single utterance; during this time the learning was switched on. This was followed by the probe phase where all stimuli were presented 50 times in randomized order, without gaps, and with the learning switched off. The output spikes from the B_2_ were counted for each stimulus and the total number of spikes recorded. This allows the SSI to be calculated for the speech-derived stimuli in the same way as for the fm sweeps in the hardware results—using the methods detailed in section 2.1.3. Sample results are shown in Figure 9.

In addition to the ssi it is possible, because these experiments can be interpreted as a set of keyword spotting trials based on spiking rate, to characterize the network as a binary classifier. The output spikes from the B_2_ neurons were counted during each stimulus, and the total number of spikes recorded; from these data we can construct the roc and hence the auc of the responses of the network.

#### 2.1.5. Learning predictions

The third set of results in section 3.3 deals with analytical predictions of what the network, either hardware or software, would learn in ideal circumstances if exposed to an arbitrary stimulus. These analytical predictions of what pattern of learning would result from exposure to a particular stimulus are based on the principle, mentioned in section 2, that the function of the B_2_ neurons is to learn correlations between activity at different times at different tonotopic channels. Calculating the strength of these correlations should therefore give us an approximation of the connectivity pattern that would result from exposure to any arbitrary stimulus.

We calculate the strength of the correlation, and hence the predicted strength of connectivity, between two network channels *x* and *y* after exposure. This can be written *C*_*x, y*_ and is calculated as the sum of the products of the stimulus activity *A*, over all times *t*, over all pairs of frequency channels *x, y*, taking in to account the time difference caused by the delays in the lateral connections Δ_*t*_, and the time difference between the pre- and post-synaptic spikes that is required by the stdp rule ϵ. The stdp rule also penalizes any activity in *y* that precedes activity in *x* thus the pattern of connectivity can be approximated by:

$$C(x,y)=\sum_{t}\left[\left(A_{x,t}\cdot A_{y,t+\Delta_{t}+\epsilon }\right)-\left(A_{x,t}\cdot A_{y,t+\Delta_{t}-\epsilon }\right)\right]$$

The value of Δ_*t*_ is a function of the channel separation between *x* and *y*, and the time taken for the activity to propagate between adjacent channels ν:

$$\Delta_{t}=|x-y|\cdot \nu$$

It is important to note that the range of effective values of ν is extremely limited in the current hardware due to the implementation of the delays using the variability of time constants that result from the fabrication of the chip (Sheik et al., 2011, 2012). However, although this limitation is taken in to account in the software model results, future hardware designs need not exhibit these limitations if the delays are implemented differently. It is partly to explore these possibilities that results in section 3.3 include examples that employ a wide range of values for ν.

**Figure 6:**
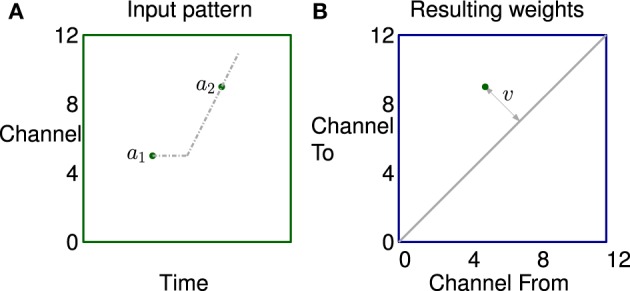
**The learning of a simple correlation in the network. (A)** Activity in a channel followed by activity in another channel some time later, represented by the dots *a*_1_ and *a*_2_ which are in channels 5 and 9 in this example. The propagation of activity through the lateral connections has a fixed offset and a velocity each represented by broken gray lines in **(A)**. **(B)** Plastic synapses connecting the neuron excited by *a*_1_ to the neuron excited by *a*_2_ are potentiated if they are on the broken gray line representing the propagation of activity. These synapses are at position (5,9) on the weight matrix shown by a dot. The distance from the diagonal *v* is proportional to the apparent sweep velocity from *a*_1_ to *a*_2_.

A simple example of how correlation in the stimulus leads to potentiation of a small set of synapses is illustrated in Figure 6. The left subfigure shows activity in a channel followed by activity in another channel some time later, represented by two dots. The propagation of activity through the lateral connections has a fixed offset and a velocity; represented by horizontal and sloping broken gray lines respectively. The right subfigure shows that the synapses connecting neurons in two channels are potentiated if they lie on the broken gray line representing the propagation.

**Figure 7:**
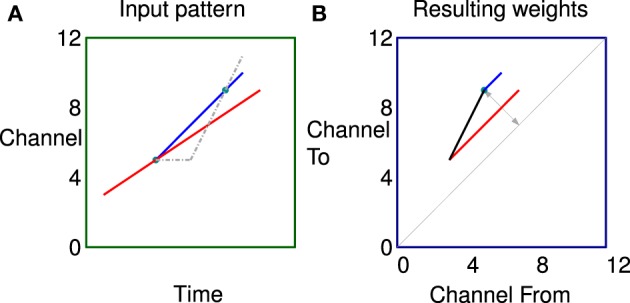
**Correlations in a more complex stimulus. (A)** Stimulus consisting of two rising tones, the two dots are in exactly the same position as Figure 6 for comparison. **(B)** The network can learn the fact that there are two sweep velocities present at the same time as indicated by the colors. Because the sweeps are linear the potentiated synapses representing the individual sweeps (red and blue) are parallel to the diagonal in the weight matrix. The black synapses are potentiated by the apparent “up” velocities between pairs of points of different colors as they diverge.

In a second more complex example shown in Figure 7 the two labeled dots are in exactly the same position as Figure 6 for comparison. In this case however the stimulus consists of two tones, both rising in frequency but with different rates and starting times. The network can learn the fact that there are two sweep velocities present at the same time as indicated by the predicted connectivity pattern. Because the sweeps are linear the potentiated synapses in red and blue are parallel to the diagonal in the weight matrix. The black synapses are potentiated by the apparent ‘up’ velocities between pairs of points of different colors as they diverge. Note, there will be no corresponding apparent “down” correlations (below the diagonal) until the sweeps are further apart because of the fixed propagation offset.

## 3. Results

### 3.1. FM sweeps

**Figure 8:**
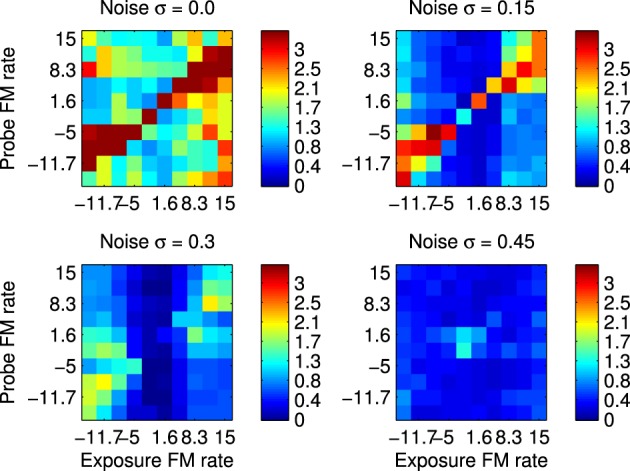
**Robustness to variation in the stimulus using hardware implementation and synthetic fm sweeps**. These plots illustrate the Stimulus Specific Information for trained network and fm sweeps using noisy stimuli. Subfigures represent increasing values of added noise σ that causes the spike pattern to added to and disrupted from the simple sweep produced by current injection in to successive channels as illustrated in Figure 2. Color scale is in bits. The maximum value is log_2_(10)≈3.32 as there are 10 classes of stimuli, interpreting each fm rate as a separate class.

The first set of results were obtained by recording spikes from silicon neurons in a hardware implementation of the network shown in Figure 1. Using the spikes recorded form the *B*_2_ neurons it is possible to calculate the ssi with respect to all the fm Probe Stimuli (ps) after using each these as Exposure Stimuli (es). These results are shown in Figure 8 which summarizes the ssi for all Exposure-Probe stimulus combinations at four noise levels. Figure 8 shows that the maximum of the ssi occurs often, but not always, at the sweep rate representing the es. This is in contrast to what we would expect if we were measuring tuning curves. The ssi measures the reduction in uncertainty, or informativeness, provided by the response which is not necessarily at the same place as the response maximum.

### 3.2. Formant tracks

**Figure 9:**
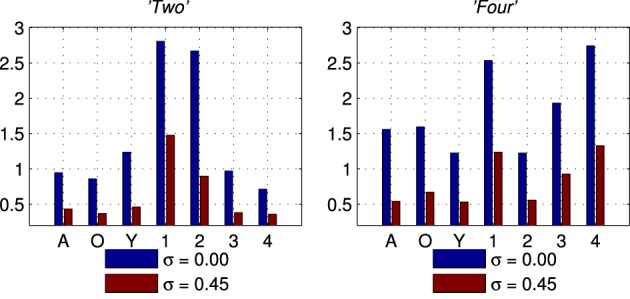
**Stimulus Specific Information results for two of the seven classes of stimuli, *“Two”* and *“Four.”*** In Blue is the no noise (σ = 0.00) condition and in Red the noisiest (σ = 0.45) condition for comparison.

The second set of results comes from the software version of the network using the simplified formant track stimuli. These results are collected in the same way as for the results in section 3.1. Figure 9 shows the ssi for two of the seven classes of Exposure Stimuli; *“Two”* and *“Four.”* The ssi values are shown for the no noise condition (σ = 0.00 in blue), and for the noisiest condition (σ = 0.45 in red). The maximum value for the ssi is log_2_(7) ≈ 2.80 there being 7 classes of stimulus. The maximum ssi is approached in the no noise condition for the es class in both cases; it is however also clear that there is information in the network response concerning all classes, not only for the class of the Exposure Stimuli. These results are representative of those obtained with all other es classes.

**Figure 10:**
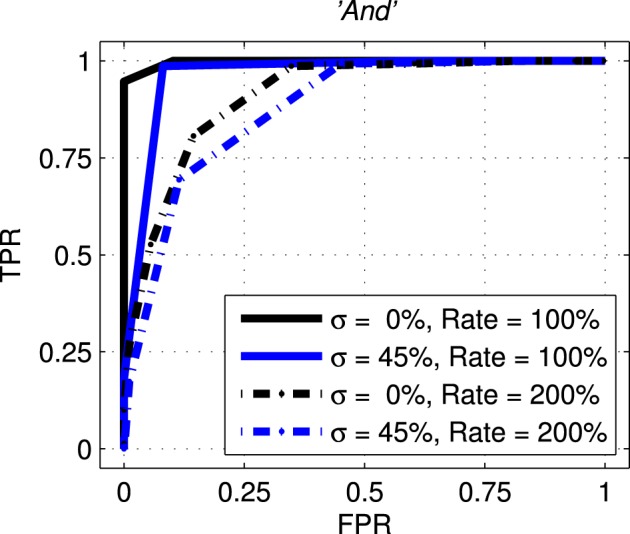
**Representative Receiver Operating curves (roc) for the network with exposure stimulus *“And.”*** roc curves plot the False Positive Rate (FPR or *fall-out*) against the True Positive Rate (TPR–sometimes called *recall*) for all detection thresholds. For clarity only four conditions are shown with two representing the best and worst case. The solid black line represents the best case with no added noise (σ = 0.00) and with the probe stimuli presented at the normal rate (Rate = 100%). The broken blue line represents the worst case across all conditions with the added noise at 45% (σ = 0.45) and the presentation rate at twice the normal rate (Rate = 200%).

The next results, shown in Figure 10, are roc curves for trials using one of the seven stimulus classes for training; *“And.”* Unlike the ssi results these figures can be obtained only by designating the Exposure Stimuli as belonging to the class to be detected by the network after training; that is treating the network as a binary classifier. Two presentation rates (Rate = 100 and 200%) combined with two noise levels (σ = 0.0 and 0.45) are shown such as to generate four conditions including the best and worst cases. Other results for this class are intermediate and this pattern is repeated for all other es classes. Full summary results from the ROC curves presented as Area Under Curve (auc) for all presentation rates and noise levels are shown, for four representative classes, in Table 1. Results for the remaining three classes are comparable.

**Table 1:** **Combined table showing Area Under Curve (auc) results for all noise conditions and all presentation rates for four of the Exposure Stimuli, *“And”,“Of”,“Yes”,“Four”***.

	**Rate**			
	**60%**	**100%**	**150%**	**200%**
**“And”**				
σ =				
0.00%	0.97	0.97	0.95	0.95
0.15%	0.94	0.95	0.96	0.93
0.35%	0.92	0.92	0.92	0.90
0.45%	0.87	0.86	0.85	0.85
**“Of”**				
σ =				
0.00%	0.88	0.93	0.86	0.84
0.15%	0.88	0.88	0.87	0.84
0.35%	0.85	0.84	0.85	0.80
0.45%	0.78	0.76	0.81	0.78
**“Yes”**				
σ =				
0.00%	0.95	0.98	0.96	0.95
0.15%	0.95	0.95	0.95	0.94
0.35%	0.92	0.92	0.91	0.90
0.45%	0.88	0.85	0.86	0.81
**“Four”**				
σ =				
0.00%	0.96	0.97	0.97	0.95
0.15%	0.95	0.96	0.96	0.92
0.35%	0.90	0.94	0.93	0.88
0.45%	0.86	0.87	0.85	0.82

*Example roc curves for the “And” stimulus can be seen in Figure 10. Other roc and auc results are comparable in all seven classes*.

### 3.3. Predicted patterns of learning

The third and final set of results shows the predicted pattern of connectivity that would result from the exposure of an idealized network to spectrographic representations derived from real sounds. These results are derived from the analytical approach described in section 2.1.5.

**Figure 11:**
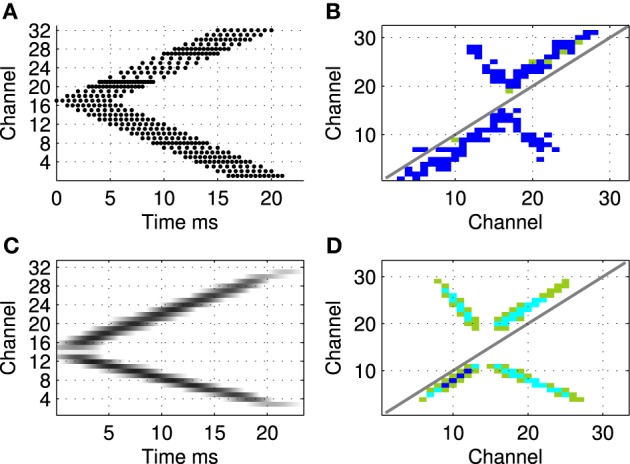
**Comparison between hardware result using a synthetic stimulus pattern (A,B) and learning prediction using a real sound file (C,D)**. Top row shows raster of synthetic exposure stimulus **(A)** and resulting network connectivity after exposure **(B)** for hardware network—these figures are taken from Sheik et al. (2011). Bottom row shows spectrogram of comparable sound file **(C)** and the analytically predicted pattern of connectivity **(D)** based on correlations in the stimulus representation as described in section 2.1.5.

First the approach is validated using sound files designed to mimic the simple patterns used in the other experiments previously reported. Figure 11 shows the predicted connectivity pattern derived from a spectrographic representation of a sound file, alongside a previously reported result from Sheik et al. (2011) using a synthetic stimulus pattern in the hardware implementation. A range of simple patterns give comparable results in hardware and software.

**Figure 12:**
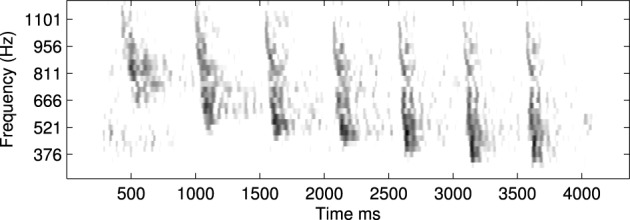
**An example of a natural communication call, in this case a Weddle Seal, shown here as a spectrogram**. This pattern was used to derive the predicted learning patterns shown in Figure 13.

**Figure 13:**
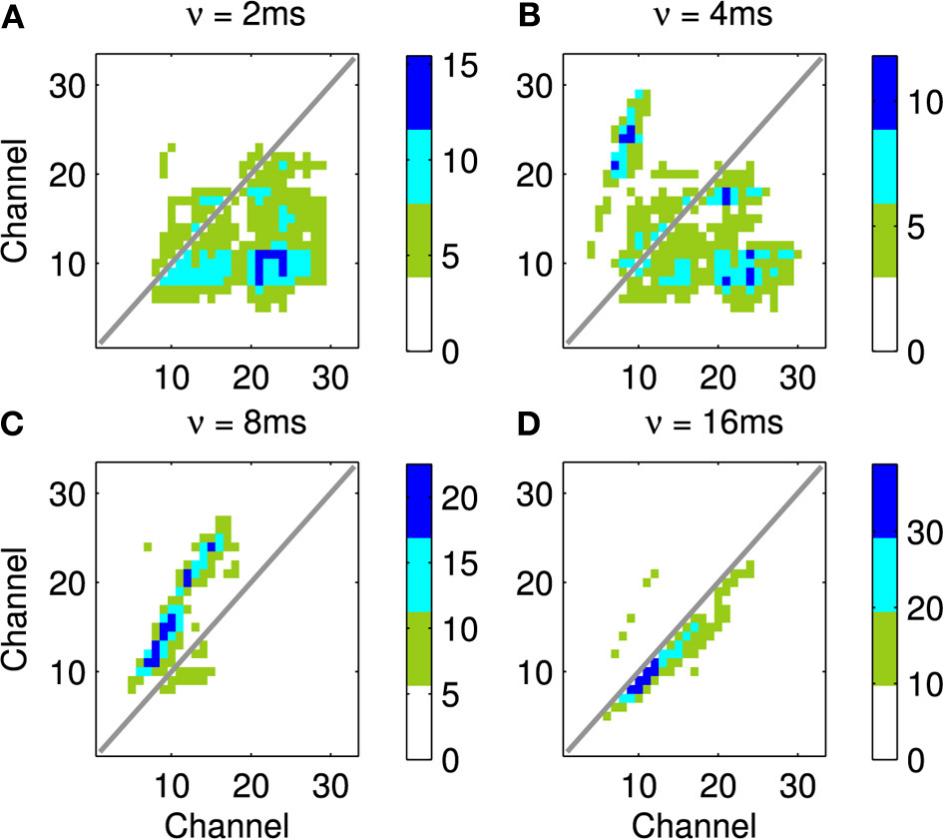
**This figure shows the predicted connectivity of the network, as Figure 11D, but for the Weddle seal call; the spectrogram for this call is shown in Figure 12**. There are four predictions based on a range of values for the propagation rate between channels. **(A–D)** show the predicted pattern of connectivity with increasing values of ν corresponding to the network adapting to correlations at progressively longer time scales. Note that in **(A)** the distance from the diagonal to the lower left local maximum represents the “chirp” fm rate. In **(C)** a clear apparent up-sweep is caused by the low frequency maxima of each chirp and the majority of the next successive chirp being at higher frequency. The **(B)** shows features of **(A,C)**. The **(D)** captures less of the dynamic nature of the stimulus.

An example of this approach using a recording of a biological communication call is shown in Figure 13. The example chosen is a recording of a call from a Weddle Seal; the cochleagraphic representation of this call can be seen in Figure 12. These results show the predicted connection patterns that would result from training a network similar to that used in the hardware and simulation experiments. However the results require a wider range of propagation rates between channels than can be achieved with the current hardware.

Four results are illustrated in Figure 13, each using a different value of ν, the time taken for activity to propagate between adjacent channels. Figure 13A shows the result with the lowest value for ν; note the emphasis on connections below the diagonal indicating down-sweeps and the distance from the diagonal to the lower left maximum of the connectivity represents the “chirp” fm rate of the successive downward sweeps in the seal call. In contrast to A the predicted connectivity in C results from an apparent up sweep. This apparent “up” activity in fact represents correlations between successive down-sweeps, that is the relationship between the maxima of each down-sweep (at low frequency) and the majority of the succeeding down-sweep at higher frequency. B contains features visible in A and C and so best characterizes the stimulus, while the longest value for ν in Figure 13D captures few, if any, of the dynamic features of the stimulus.

## 4. Discussion and conclusion

The results presented here show that the previously published results and approach (Sheik et al., 2011) are not limited to simple stereotypical stimuli and, even in this highly challenging arena, that there is scope for implementing systems that are robust to realistic signal variability. The stimuli used in these studies exhibit a range of different spectro-temporal properties, are presented continuously rather than in isolation, exhibit wide variability due to added noise, and have a variable presentation rate. All of these complications, and distortions, represent a substantial challenge and are necessary prerequisites to the development of systems that can deployed in real situations.

In section 2.1.5 we discuss how the network is capable of simultaneously representing the position, rate of change, and spectral distance (and to a more limited extent temporal distance) between features in the stimuli. Adaptive sensitivity to all of these has been demonstrated in hardware and software. The robustness in the system is derived from the fact that, although noise and variable presentation rate alter or degrade these patterns of features, it requires either or both types of variability to be present to a very large degree for the degradation to cause the correlations to be masked completely.

We have introduced an information theoretic characterization of the performance of the network, the SSI, based on the variability of the stimuli and the consequent range of responses to a single stimulus class. This represents a method of quantifying the performance of a hardware system that has not been previously reported in an engineering context, but has direct parallels in physiological measurements. The substitution of an information theoretic measure for a classifier is deliberate, because it focusses on the information present in the response rather than the design or performance of the classifier. Our results, summarized in Figures 8 and 9 indicate that the adaptation of the network to the formative stimulus produces a differential response that is informative with respect to all classes.

Sensory stimuli, in particular auditory stimuli, contain both short and long range temporal correlations. The techniques currently employed in the hardware implementation primarily address correlations only over time scales of the order of synaptic or membrane time constants, up to those represented by the propagation of excitation to adjacent regions. However we have shown that the principles embodied in the network could be extended to longer time scales making it feasible to build systems capable of adapting to complex stimuli, such as animal communication calls. In hardware, longer time scales could be addressed using many levels of recurrence between widely separated layers, as is observed in the mammalian auditory system. Alternatively, from a pragmatic perspective, it could be tackled with working memory and neuromorphic implementations of state machine based approaches (Neftci et al., 2013).

Alongside our previously reported results (Sheik et al., 2011) we pointed out that in order to be useful, the properties of the neuromorphic system we described would have to be validated against noise and other variations in the stimulus, and to be shown to work with more realistic stimuli. We also promised to go beyond the demonstration of emergent sensitivity to a stimulus parameter, and to quantify the increase in acuity in information-theoretic terms; thus providing a basis for the quantitative comparison of networks, connectivity patterns, and learning strategies in the future. In this work we have made significant progress in all of these aims. The approach has been shown to be capable of handling considerable stimulus variation, changes in presentation rate, and the increased complexity of stimulus. Had it fallen at any of these hurdles then the feasibility of the approach would have been called in to question. It is clear, then, that each of these new results is evidence that the approach could lead to a neuromorphic subsystem engineered for dynamic pattern recognition in real world applications.

### Conflict of interest statement

The authors declare that the research was conducted in the absence of any commercial or financial relationships that could be construed as a potential conflict of interest.
